# The tip of the iceberg: a giant pelvic atypical lipoma presenting as a sciatic hernia

**DOI:** 10.1186/1477-7819-4-33

**Published:** 2006-06-21

**Authors:** Richard JE Skipworth, Graeme HM Smith, Ken J Stewart, David N Anderson

**Affiliations:** 1 Department of General Surgery, St. John's Hospital at Howden, Livingston, NHS Lothian – University Hospitals Division, UK

## Abstract

**Background:**

This case report highlights two unusual surgical phenomena: lipoma-like well-differentiated liposarcomas and sciatic hernias. It illustrates the need to be aware that hernias may not always simply contain intra-abdominal viscera.

**Case presentation:**

A 36 year old woman presented with an expanding, yet reducible, right gluteal mass, indicative of a sciatic hernia. However, magnetic resonance imaging demonstrated a large intra- and extra-pelvic fatty mass traversing the greater sciatic foramen. The tumour was surgically removed through an abdomino-perineal approach. Subsequent pathological examination revealed an atypical lipomatous tumour (synonym: lipoma-like well-differentiated liposarcoma). The patient remains free from recurrence two years following her surgery.

**Conclusion:**

The presence of a gluteal mass should always suggest the possibility of a sciatic hernia. However, in this case, the hernia consisted of an atypical lipoma spanning the greater sciatic foramen. Although lipoma-like well-differentiated liposarcomas have only a low potential for recurrence, the variable nature of fatty tumours demands that patients require regular clinical and radiological review.

## Background

We report the case of a young woman in whom a lipoma-like well differentiated liposarcoma presents as a sciatic hernia. This case therefore highlights two unusual surgical phenomena, and illustrates the need for clinicians to be aware that hernias may not always simply contain intra-abdominal viscera. We also review the medical literature surrounding lipoma-like well differentiated liposarcoma and sciatic hernias.

## Case report

**Figure 1:**
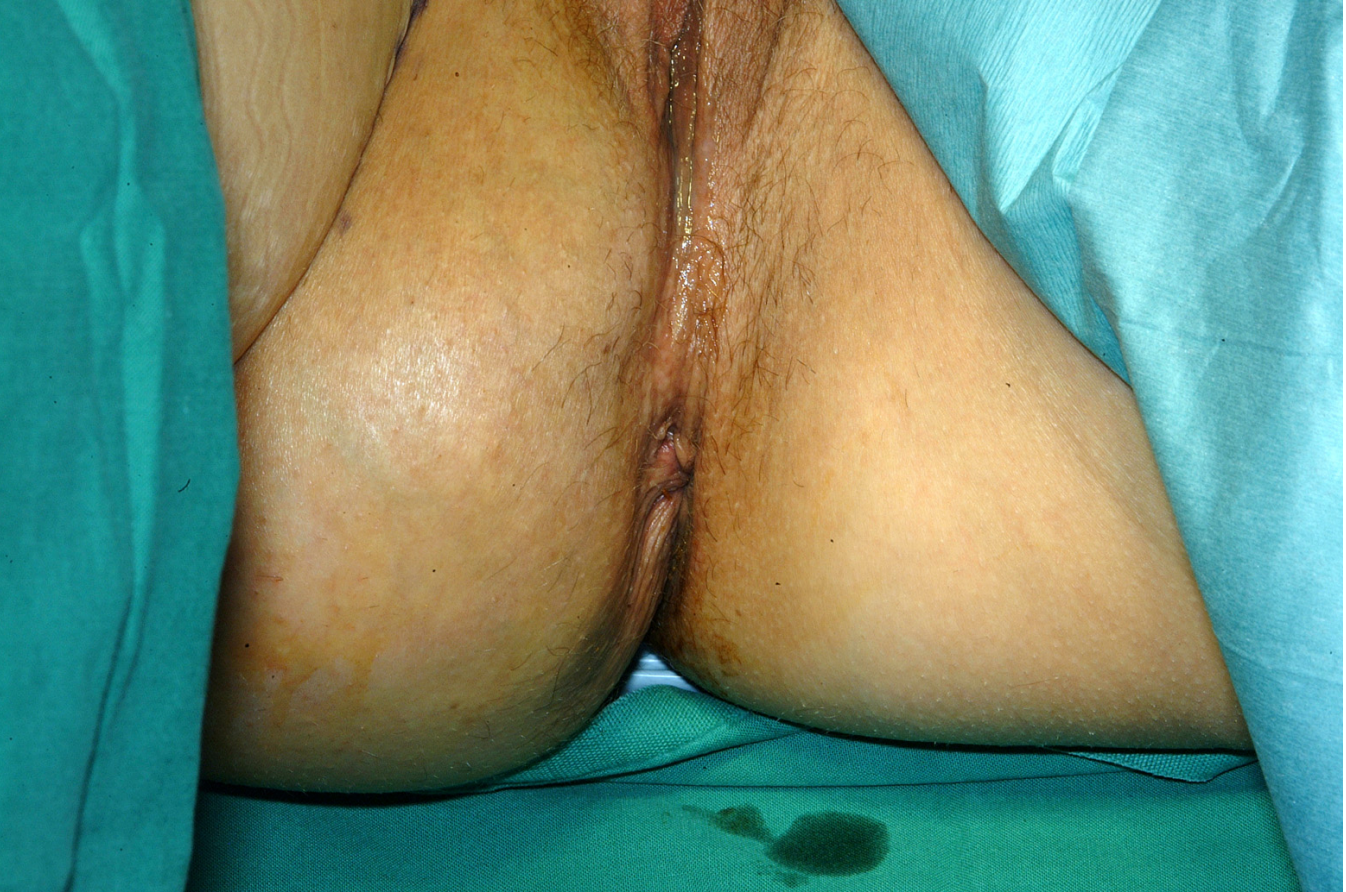
Photograph demonstrating obvious swelling in right gluteal area.

A 36 year old woman was referred by her family physicians with a 3 year history of a large lump on her right buttock (Fig. 1). She had noted a recent increase in its size, such that it hung down creating a visible bulge in the seat of her trousers. She had no other past medical history of note, and she took no medications.

On examination, she had a large swelling at the ischial area of her right buttock that, in most respects, was consistent with a typical subcutaneous lipoma. However, it had the unusual feature that it was reducible in a similar way to a sciatic hernia.

## Imaging

An MRI scan demonstrated that the lesion was not simply a subcutaneous lipoma.

It showed a well-defined 14 cm × 8 cm × 8 cm maximum diameter mass that traversed the greater sciatic foramen through the infra-piriformis area. It lay partly within the pelvis and partly within the right ischiorectal fossa and upper thigh.

The tumour was homogenously iso-intense with fat except for a small focus of low signal intensity at its superior margin. There was no abnormal signal on the STIR sequence, which demonstrated normal fat suppression.

**Figure 2:**
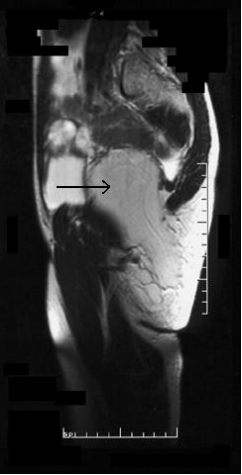
Sagittal T1-weighted MRI section (arrow indicating tumour).

**Figure 3:**
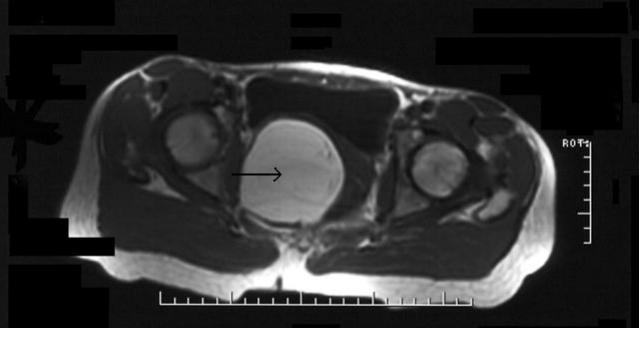
Transverse T1-weighted MRI section (arrow indicating tumour).

The size and situation of the mass displaced the pelvic organs, resulting in the bladder being pushed anteriorly, the uterus superiorly and the rectum to the left. Inferiorly, the mass extended through the ischio-rectal fossa, medial to the ischial tuberosity, to come to lie in the subcutaneous fat medial to sartorius and the hip adductors (Fig. 2 and 3). Except for its size and deep position, there were no sinister features, although an atypical lipoma or well differentiated liposarcoma could not be excluded.

## Surgery

**Figure 4:**
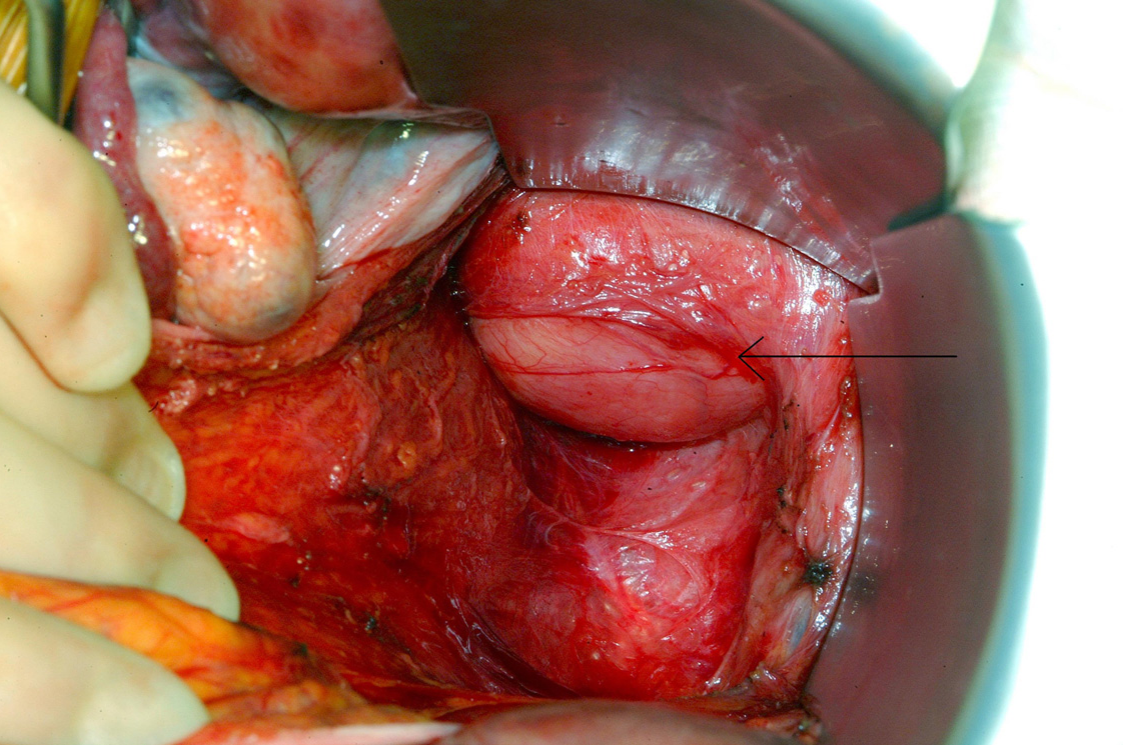
View of the tumour within the pelvis (arrow demonstrating tumour).

**Figure 5:**
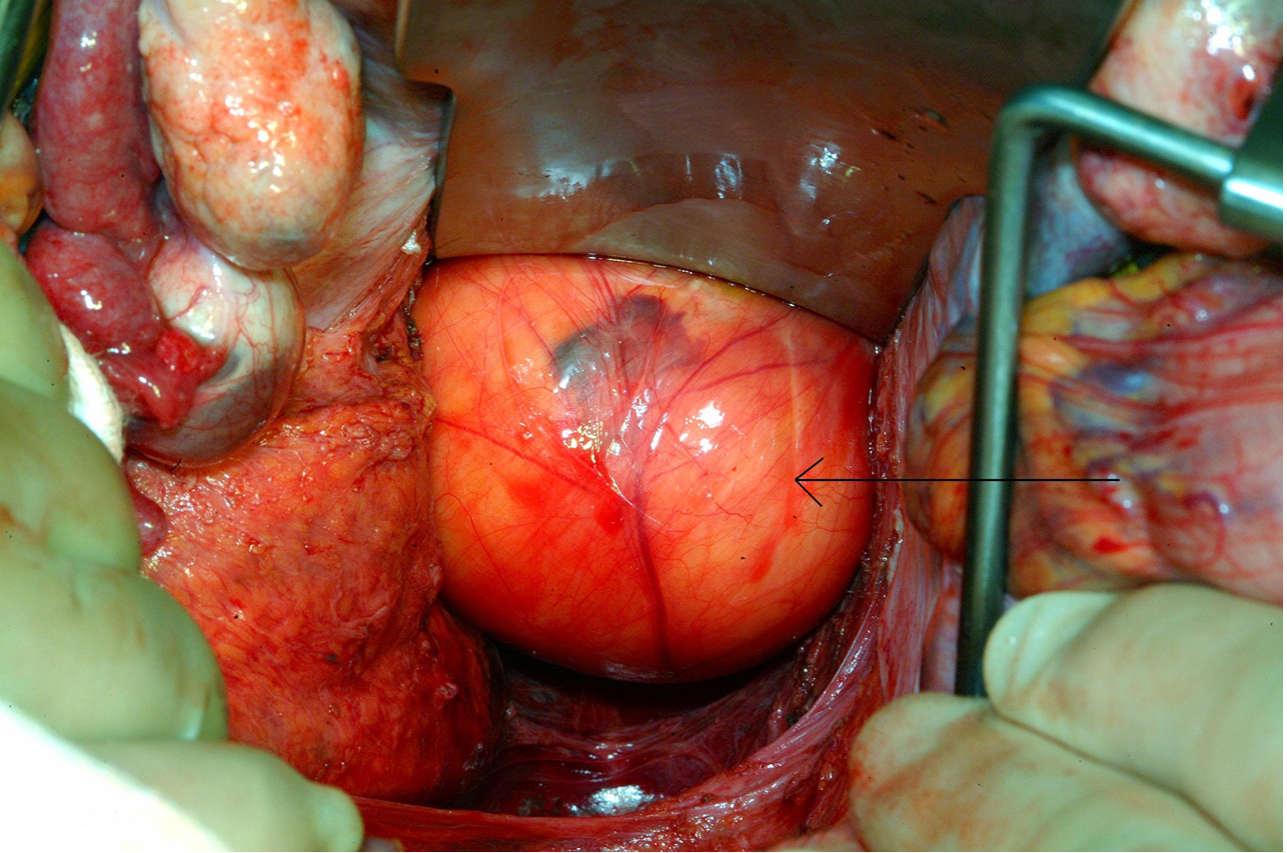
View of the tumour within the pelvis (arrow demonstrating tumour).

**Figure 6:**
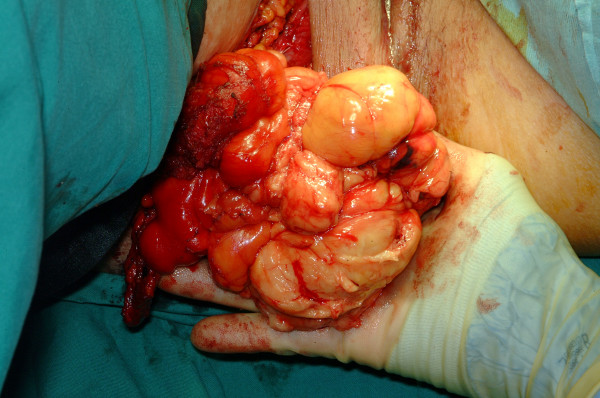
Delivery of the tumour via the perineal wound.

After discussion with the local sarcoma multi-disciplinary team, the plastic and general surgeons removed the tumour in a joint procedure. To fully reduce the tumour back into the pelvic cavity (via the greater sciatic foramen) and maintain its position there, an assistant was employed to exert external manual pressure on the right buttock throughout the procedure. Tumour dissection was then performed through an abdomino-perineal approach (Fig. 4 and 5). A 4 cm × 4 cm right sciatic defect was identified. Piriformis muscle was not easily identified due to atrophy and fibrosis. Tumour removal was performed through the perineal wound (Fig. 6). A large omental patch was fully mobilised from the greater omentum and was used to plug the sciatic defect. In this way, the use of prosthetic mesh was avoided.

## Pathology

**Figure 7:**
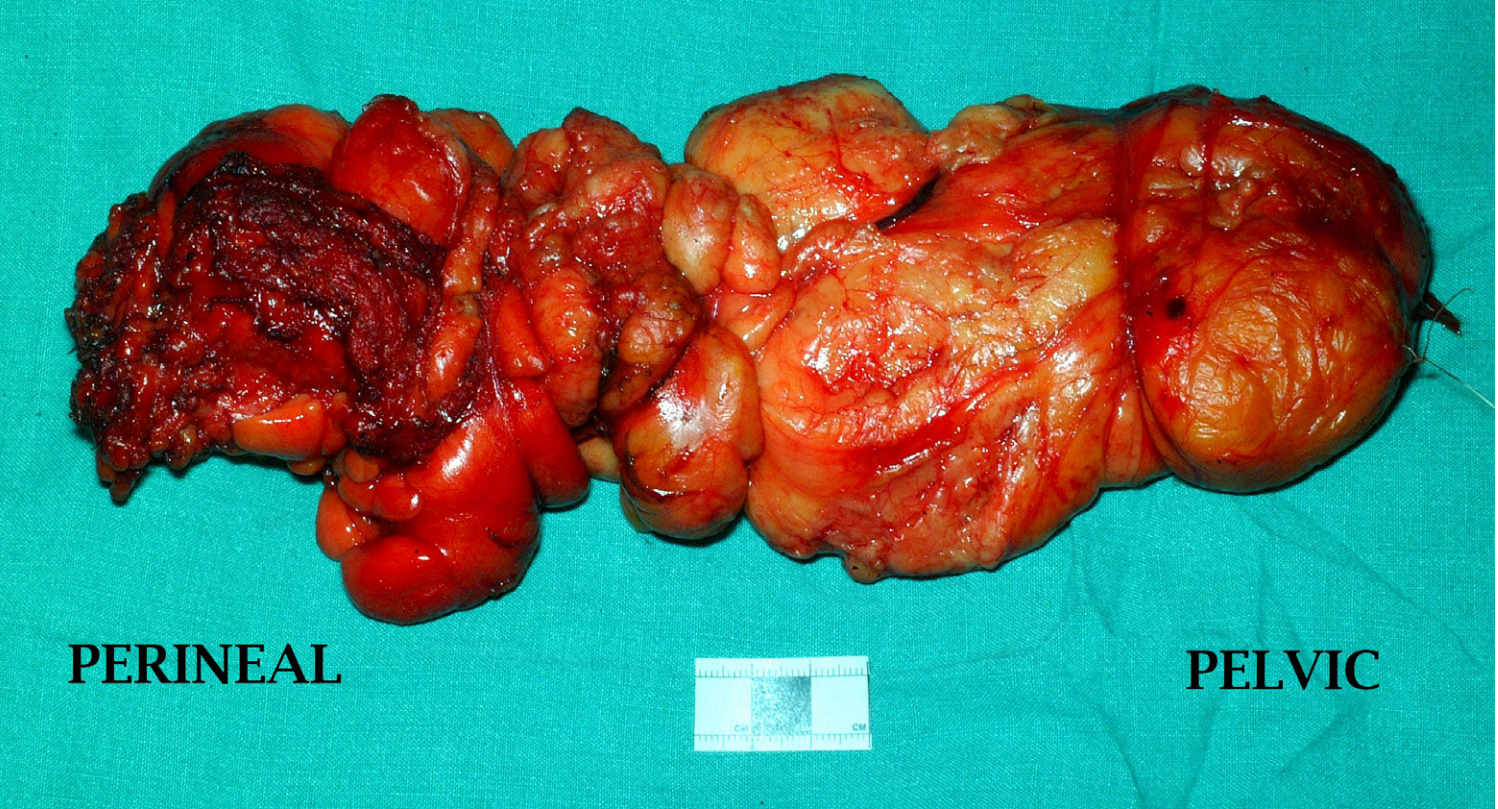
Gross appearance of the tumour after excision.

The tumour was a lobulated mass of adipose tissue measuring 230 mm × 180 mm × 40 mm (Fig. 7). Histopathological examination revealed mild nuclear enlargement and variation throughout, but no significant atypia, hyperchromasia or mitotic activity.

These features were in keeping with an atypical lipomatous tumour (synonym: lipoma-like well-differentiated liposarcoma) with low potential for recurrence.

## Follow-Up

The patient remains under the care of the plastic surgeons. She has remained well for 2 years following her surgery and follow-up CT scans have not demonstrated recurrence.

## Discussion

Sciatic hernia is an extremely rare variety of hernia. Following its initial description by Papen in 1750, there have been less than a hundred case reports in the medical literature [1]. It occurs in both children and adults, with a female predomination in the adult cases [2]. There are two forms – greater sciatic (synonym: gluteal) and lesser sciatic (synonym: sciatic). It has been postulated that neuromuscular disease, hip pathology, or other locomotor disturbances of the lower limb may predispose persons to herniation as a result of piriformis muscle atrophy [3]. Other theories have suggested adhesions, congenital anomalies [4] or fascial defects [2] as possible causes.

Sciatic hernias may present as a gluteal mass, as a rare cause of sciatica (following compression of the sciatic nerve) [5], or with complications of their contents. Such contents have included small bowel (sometimes leading to obstruction) [6,7], the ureter or bladder (causing urinary tract symptoms) [3,4], ovaries and fallopian tubes (causing pelvic pain syndromes) [8], colon, omentum, and Meckel's diverticulum. To our knowledge, the only previous description of a lipoma herniating through the sciatic foramen occurred in 1964 [9].

Symptomatic hernias should be surgically repaired as soon as possible (with mesh, if required), through either a transabdominal or transgluteal approach [10,11].

Well-differentiated liposarcoma accounts for about 40% to 45% of all liposarcomas [12], and therefore represents the larger subgroup of adipocytic malignancies. It tends to occur equally in the retroperitoneum or the limb, followed by the paratesticular area and the mediastinum, with a peak incidence between the fifth and seventh decades.

Well-differentiated liposarcoma is further subdivided into the lipoma-like (adipocytic), sclerosing, inflammatory and spindle cell subtypes, of which the first two are by far the commoner. Our case was an example of the lipoma-like variant of well-differentiated liposarcoma. These tumours, as they sound, are composed mainly of univacuolated adipocytes showing some variation in size and shape, associated with only scattered multivacuolated lipoblasts (the presence of which are an absolute prerequisite for the diagnosis of liposarcoma) and occasional hyperchromatic spindle or stellate cells, which are often found within dense fibrous septa [13]. It has been suggested that these thick septa, which contain skeletal muscle elements, may be one method of differentiating malignant tumours from simple lipomas, on fat-suppressed T1-weighted MR images after gadolinium (III) diethyltriaminepentaacetic acid (Gd-DTPA) administration [14]. Other features that suggest malignancy include increased patient age, large lesion size, presence of nodular/and or globular or non-adipose mass-like areas, and decreased percentage of fat composition [15].

Cytogenetically, well-differentiated liposarcomas appear to be relatively homogenous, exhibiting characteristic ring, as well as giant marker chromosomes, containing amplified genetic material derived from the 12q 13–15 chromosome region [16].

It is important that all well-differentiated liposarcomas are extensively sampled, both to ensure identification of lipoblasts (hence avoiding confusion with, for example, intramuscular lipoma), and to exclude the presence of a dedifferentiated element. Such an area of dedifferentiation confers a much worse prognosis, with up to a 50% chance of metastasis [13]. Dedifferentiation is largely a time-dependent phenomenon that occurs in sites in which there is a high likelihood for clinical persistence of disease (e.g. the retroperitoneum) [16]. One study demonstrated that the time interval between diagnosis and dedifferentiation might be as long as 18 years [17]. However, the progression of the disease following dedifferentiation may be highly variable and probably depends on a number of factors, including the amount of dedifferentiation and type of therapy.

The classification of these tumours has been a source of debate for pathologists in recent years, but the current trend is to classify all pure well-differentiated cases arising in a limb as atypical lipoma, since, although they commonly recur, they never metastasise and therefore wide excision should be curative. Liposuction alone has been advocated as a possible treatment for these tumours [18]. However, tumours of the retroperitoneum, irrespective of microscopic appearance, have a five-year survival of approximately 35%, owing to their usual incomplete excision and repeated local recurrence with involvement of local structures [13].

In this particular case, we have employed CT as the radiological modality of follow-up. However, this decision was largely based on the availability of local resources and we would otherwise usually recommend MRI. As lipoma-like well-differentiated liposarcoma have only a low potential for recurrence, we would suggest that follow-up MRI need only be performed annually or when new symptoms evolve.

## Competing interests

The author(s) declare that they have no competing interests.

## Authors' contributions

RJES is a surgical trainee who was involved in this patient's management. He was also involved in drafting and revising the manuscript.

GHMS is a surgical trainee who was involved in this patient's management. He was also involved in drafting and revising the manuscript.

KJS was the Plastic Surgery Consultant in charge of this patient's surgical management. He was also involved in drafting and revising the manuscript.

DNA was the General Surgery Consultant in charge of this patient's surgical management. He was also involved in drafting and revising the manuscript.

All authors read and approved the final manuscript.
